# Turing instability mechanism of short-memory formation in multilayer FitzHugh-Nagumo network

**DOI:** 10.3389/fpsyt.2023.1083015

**Published:** 2023-03-27

**Authors:** Junjie Wang, Jianwei Shen

**Affiliations:** ^1^School of Mathematics and Statistics, Zhengzhou University, Zhengzhou, China; ^2^School of Mathematics and Statistics, North China University of Water Resources and Electric Power, Zhengzhou, China

**Keywords:** FHN model, short-term memory, multilayer network, Turing pattern, delay, Hopf bifurcation, noise

## Abstract

**Introduction:**

The study of brain function has been favored by scientists, but the mechanism of short-term memory formation has yet to be precise.

**Research problem:**

Since the formation of short-term memories depends on neuronal activity, we try to explain the mechanism from the neuron level in this paper.

**Research contents and methods:**

Due to the modular structures of the brain, we analyze the pattern properties of the FitzHugh-Nagumo model (FHN) on a multilayer network (coupled by a random network). The conditions of short-term memory formation in the multilayer FHN model are obtained. Then the time delay is introduced to more closely match patterns of brain activity. The properties of periodic solutions are obtained by the central manifold theorem.

**Conclusion:**

When the diffusion coeffcient, noise intensity *np*, and network connection probability *p* reach a specific range, the brain forms a relatively vague memory. It is found that network and time delay can induce complex cluster dynamics. And the synchrony increases with the increase of *p*. That is, short-term memory becomes clearer.

## 1. Introduction

In 1952, Alan Hodgkin and Andrew Huxley developed the famous Hodgkin-Huxley (HH) model based on nerve stimulation potential data of squid. Due to the high dimension and computational complexity of the HH model, Richard FitzHugh and J.Nagumo simplified the HH model and established the FHN model. In the actual nerve conduction process, there is a certain time delay in signal transmission, which caused a lot of research on the FHN model with time delay. Wang et al. studied bifurcation and synchronization (1), bifurcation structure (2), Fold-Hopf bifurcation (3), periodic oscillation (4), and global Hopf bifurcation (5) of coupled FHN model with time delay. Yu et al. (6) found that the noise level can change the signal transmission performance in the FHN network, and the delay can cause multiple stochastic resonances. Gan et al. (7) also found that appropriate delay can induce stochastic resonances in FHN scale-free networks and devoted themselves to extending the range of stochastic resonance on complex neural networks. Zeng et al. (8) found that, unlike noise, the system undergoes a phase transition as the time delay increases. Bashkirtseva and Ryashko analyzed the excitability of the FHN model using the stochastic sensitivity function technique and proposed a new method for analyzing attractors (9). In addition, it is found that there are very complex dynamic phenomena in the FHN model. Rajagopal et al. (10) studied chaos and periodic bifurcation diagrams under different excitation currents and found that the dynamic behavior of the nodes alters dramatically after the introduction of Gaussian noise. Iqbal et al. (11) studied robust adaptive synchronization of a ring-coupled uncertain chaotic FHN model and proposed a scheme to synchronize the coupled neurons under external electrical stimulation. Feng et al. studied the influence of external electromagnetic radiation on the FHN model. And they found that periodic, quasi-periodic, and chaotic motions would occur in different frequency intervals when the external electromagnetic radiation was in the form of a cosine function (12).

In the same year, the HH model was proposed, Turing discovered that a stable uniform state would become unstable under certain conditions in a reaction-diffusion system, which attracted a lot of attention and was introduced into various fields. Liu et al. found that cross-diffusion could lead to Turing instability of periodic solutions (13, 14). Lin et al. analyzed the conditions of Turing-Hopf bifurcation and the spatiotemporal dynamics near the bifurcation point in diffusion neural networks with time delay (15). Mondal et al. studied the dynamical behaviors near the Turing-Hopf bifurcation points of the neural model. And they found that collective behaviors may be related to the generation of some brain pathologies (16). Qu and Zhang studied the conditions required for various bifurcations in the FHN diffusion system under Neumann boundary conditions and extended them to coupled FHN model (17). With the boom of complex networks in recent years, many scholars have begun to study the Turing pattern under the network (18–22). Ren et al. extended these studies to multilayer networks (23, 24). Moreover, Tian et al. investigated pattern and Hopf bifurcation caused by time delay in the Small-World network, Barabasi-Albert free-scale network, and Watts-Strogatz network (25, 26). These studies take the pattern problem to a new level.

Researchers are keen to study some characteristic behaviors of the brain from the perspective of the network because the brain is a complex network system with hierarchical and modular structures (27). Neurons generate complex cluster dynamic behaviors through synaptic coupling to form brain function. Neurons with similar connection patterns usually have the same functional attributes (28). Experiments have shown that neurons far apart can fire simultaneously when the brain is stimulated and that this phenomenon persists when neurons are in the resting state. One of the brain's basic functions is remembering information, which can be a sensory stimulus or a text (29). The principle of memory formation is very complex and is still being explored. A classic view is that the realization of short-term memory in the brain depends on fixed point attractors (30, 31). Memory storage is maintained by the continuous activity of neurons, which persists even after the memory stimulus has been removed (32, 33). Goldman showed the fundamental mechanisms that generate sustained neuronal activity in feedforward and recurrent networks (33). Neurons release neurotransmitters that direct human activity when the brain receives the information. However, due to the noise and the existence of inhibitory neurons, information processing cannot always be synchronized in time, which leads to a certain delay in the recovery time of action potentials (34). And Yu et al. found that the delay will affect the transmission performance of sub-threshold signal and induce various chaotic resonances in coupled neural networks (35).

The state of neurons can be represented by patterns. The pattern no longer looks so smooth when the brain stores short-term memory. There is synchrony in the activity of neurons. In pattern dynamics, synchronization can be induced by Turing instability. Scholars have built various mathematical models and analyzed neurons using Turing dynamic theory to understand the mechanism of memory formation. Zheng et al. studied the effect of noise on the bistable state of the FHN model and explained the biological mechanism of short-term memory by the pattern dynamics theory (36). They also studied the conditions of Turing pattern generation in the Hindmarsh-Rose (HR) model and found that collected current and outgoing current greatly influenced neuronal activity and used this to explain the mechanism of short-term memory generation (37). Wang and Shi proposed the time-delay memristive HR neuron model, found multiple modes and coherence resonance, and speculated that it might be related to the memory effect of neurons (38). We study the FHN model under a multilayer network to get closer to the actual brain structure. The biological mechanism of short-term memory generation is explained by the pattern characteristics of the model. The article is structured as follows. In the next section, firstly, the stability of the equilibrium point in the FHN model is analyzed. Then the sufficient conditions for the Turing instability of the FHN model on the Cartesian product network are found using the comparison principle. Finally, the properties of periodic solutions in FHN multilayer networks are studied using the center manifold theorem. Explaining the mechanism of short-term memory by numerical simulation in Section 3.

## 2. Description of the FHN model

We consider the general FHN model


(1) $$\begin{array}{l} \frac{du}{dt}=c\left(u-u^{3}/3-av+I\right),\\ \frac{dv}{dt}=c\left(bu-v+d\right), \end{array}$$

Where *u* is membrane potential, which is a fast variable, and *v* is recovery variable, which is a slow variable. *I* is the external input current. *a, b* represent respectively the intensity of action from *v* to *u* and from *u* to *v*. And the parameters *c* ≠ 0, *d* are constants. The equilibrium point of the system (Equation 1) satisfies *u*^3^ + 3(*ab* − 1)*u* + 3(*ad* − *I*) = 0. Therefore, we have the following conclusion.

**Lemma 1** Let $\varpi =\frac{-1+\iota \sqrt{3}}{2}$ and $\varrho =\frac{3}{2}\left(ad-I\right)$, in which *ι* is the imaginary unit. The influence of parameters on the number of equilibrium of the system (Equation 1) is as follows.

(i) When *ab* − 1 = *ad* − *I* = 0, the equation has triple zero roots and the trace of the system (1) at that point is constant 0.
(ii) When Δ = ϱ^2^ + (*ab* − 1)^3^ > 0, the equation has only one real root $\sqrt[{3}]{-\varrho +\sqrt{\Delta }}+\sqrt[{3}]{-\varrho -\sqrt{\Delta }}$.
(iii) When Δ = 0, *ab* ≠ 1 and *ad* ≠ *I*, the equation has two real roots $-2\sqrt[{3}]{\varrho }$ and $\sqrt[{3}]{\varrho }$, and the determinant at the second root $\sqrt[{3}]{\varrho }$ of system (1) is always 0.
(iv) When Δ < 0, the equation has three unequal real roots $\sqrt[{3}]{-\varrho +\sqrt{\Delta }}+\sqrt[{3}]{-\varrho -\sqrt{\Delta }}$, $\varpi \sqrt[{3}]{-\varrho +\sqrt{\Delta }}+\varpi^{2}\sqrt[{3}]{-\varrho -\sqrt{\Delta }}$, $\varpi^{2}\sqrt[{3}]{-\varrho +\sqrt{\Delta }}+\varpi \sqrt[{3}]{-\varrho -\sqrt{\Delta }}$.

Let U^*^ = (u^*^, v^*^) be the equilibrium point of the system (1). By coordinate transformation *ū* = *u*(*t*)−u^*^, $\bar{v}=v\left(t\right)-v^{*}$, we get the following equivalent system. For convenience, *u*(*t*), *v*(*t*) are still used to denote *ū*, $\bar{v}$,


(2) $$\begin{array}{l} \frac{du}{dt}=a_{11}u+a_{12}v+f\left(u\right),\\ \frac{dv}{dt}=a_{21}u+a_{22}v, \end{array}$$

where $a_{11}=c\left(1-u^{{*}^{2}}\right)$, *a*_12_ = −*ac*, *a*_21_ = *bc*, *a*_22_ = −*c*, $f\left(u\right)=-u^{*}u^{2}-\frac{u^{3}}{3}$. The corresponding determinant $\Delta_{0}=c^{2}\left(u^{{*}^{2}}+ab-1\right)$ and the trace $Tr_{0}=-cu^{{*}^{2}}$. By the Routh-Hurwitz criterion, the equilibrium (0, 0) of the system (Equation 2) is stable if and only if (H 1) holds,


(H 1) $$\begin{array}{l} cu^{{*}^{2}}>0\text{ and }u^{{*}^{2}}+ab-1>0. \end{array}$$

### 2.1. FHN model on Cartesian product network

Now we discuss the effect of the Cartesian product networks on the stability of the equilibrium point (0, 0). Two networks *R* and *E* with *n*_*r*_ and *n*_*e*_ nodes are given, respectively. $\left(L^{R}\right)=A^{R}-{\left(k_{i}\delta_{ij}\right)}^{R}$ ($\left(L^{E}\right)=A^{E}-{\left(k_{i}\delta_{ij}\right)}^{E}$) is the Laplacian matrix of the network *R* (*E*). *A* is the adjacency matrix of the network. And *k*_*i*_ denotes the degree of the *i*th node. δ_*ij*_ satisfies, δ_*ij*_ = 1 when node *i* has an edge with node *j*; δ_*ij*_ = 0 when there is no edge. By using the Kronecker product, we can get the Cartesian product network *R*□*E* (□ stands for multilayer network), which has *n*_*r*_*n*_*e*_ nodes. Then the Laplacian matrix of *R*□*E* is denoted as


$$L^{R□E}=L^{R}⊗{𝕀}_{n_{e}}+{𝕀}_{n_{r}}⊗L^{E},$$

and the eigenvalues of *R*□*E* are of form


$$\Lambda_{\alpha \beta }^{R□E}=\Lambda_{\alpha }^{R}+\Lambda_{\beta }^{E},\;\alpha \in \left\{1,\cdot \cdot \cdot ,n_{r}\right\},\;\beta \in \left\{1,\cdot \cdot \cdot ,n_{e}\right\}.$$

A general FHN model on Cartesian product network can be expressed as


(3) $$\begin{array}{l} \frac{du_{re}}{dt}=a_{11}u_{re}+a_{12}v_{re}+f\left(u_{re}\right)+{\text{L}}_{u}u_{re},\\ \frac{dv_{re}}{dt}=a_{21}u_{re}+a_{22}v_{re}+{\text{L}}_{v}v_{re}, \end{array}$$

Where *r* ∈ {1, ···, *n*_*r*_}, *e* ∈ {1, ···, *n*_*e*_}. The Laplacian operator L_*u*_ is


$${\text{L}}_{u}=D_{u}^{R}L^{R}⊗{𝕀}_{n_{e}}+D_{u}^{E}{𝕀}_{n_{r}}⊗L^{E}.$$

$D_{u}^{R},\;D_{v}^{R}$ ($D_{u}^{E},\;D_{v}^{E}$) are the diffusion coefficients of the network *R* (*E*). Notice that $\left(L^{R}⊗{𝕀}_{n_{e}}\right)\left(u_{re}\right)=\left(L^{R}u_{r}^{R},u_{e}^{E}\right)=\underset{r^{\prime }}{\sum }L_{rr^{\prime }}^{R}u_{r^{\prime }e}$, and similarly, we can get ${𝕀}_{n_{r}}⊗L^{E}$. For L_*v*_*v*_*re*_, we can get similar result. Expanding *u*_*re*_ and *v*_*re*_ in Fourier space, we can obtain linearized equation for equation (3),


(4) $$\begin{array}{l} \frac{du_{re}}{dt}=a_{11}u_{re}+a_{12}v_{re}+\left(D_{u}^{R}\Lambda_{\alpha }^{R}+D_{u}^{E}\Lambda_{\beta }^{E}\right)u_{re},\\ \frac{dv_{re}}{dt}=a_{21}u_{re}+a_{22}v_{re}+\left(D_{v}^{R}\Lambda_{\alpha }^{R}+D_{v}^{E}\Lambda_{\beta }^{E}\right)v_{re}. \end{array}$$

**Lemma 2 Comparison principles** Consider the ODE


(A 1) $$\begin{array}{l} \frac{d^{2}S}{dt}+P\left(t\right)\frac{dS}{dt}+Q\left(t\right)S=0, \end{array}$$

and suppose that there exists some Φ(*t*) such that


(A 2) $$\begin{array}{l} Q\left(t\right)\leq -\frac{1}{\Phi }\frac{d^{2}\Phi }{dt}-\frac{1}{\Phi }\frac{d\Phi }{dt}P\left(t\right),\;\forall t\in \text{Ω}. \end{array}$$

If (A 2) holds, then the fundamental solution *S*(*t*) of (A 1) satisfies |*S*| ≥ Φ(*t*) for all *t* ∈ Ω. In particular, *S*(*t*) has an exponential growth rate on Ω if *Q*(*t*) < 0 for all *t* ∈ Ω.

The proof of the lemma is divided into two cases. Let's discuss it first at the boundary, and then prove it on the inside by using the properties of the Riccati equation. The detailed proof can be seen in Van Gorder (39).

**Theorem 1** Assume that (H 1) holds.


(H 2) $$\begin{array}{l} \begin{array}{l} \Delta_{0}+\Lambda_{\beta }^{E}\left(a_{22}D_{u}^{E}+a_{11}D_{v}^{E}\right)+\Lambda_{\alpha }^{R}\left(a_{22}D_{u}^{R}+a_{11}D_{v}^{R}\right)\\ +{\left(\Lambda_{\beta }^{E}\right)}^{2}D_{u}^{E}D_{v}^{E}+\Lambda_{\beta }^{E}\Lambda_{\alpha }^{R}\left(D_{u}^{E}D_{v}^{R}+D_{u}^{R}D_{v}^{E}\right)+{\left(\Lambda_{\alpha }^{R}\right)}^{2}D_{u}^{R}D_{v}^{R}<0. \end{array} \end{array}$$

If (H 2) holds, then (0, 0) for the system (Equation 3) is linearly unstable.

**Proof** We consider the Equation (4). Separating *v*_*re*_ from the first equation of Equation (4), we can obtain


$$\begin{array}{l} v_{re}=\frac{u_{re^{\prime }}-a_{11}u_{re}-\left(D_{u}^{R}\Lambda_{\alpha }^{R}+D_{u}^{E}\Lambda_{\beta }^{E}\right)u_{re}}{a_{12}}. \end{array}$$

Putting it into the second equation of Equation (4), we can obtain a second-order ODE about *u*_*re*_,


$$\begin{array}{l} \begin{array}{l} u_{re^{\prime \prime }}-\left[Tr_{0}+\Lambda_{\beta }^{E}\left(D_{u}^{E}+D_{v}^{E}\right)+\Lambda_{\alpha }^{R}\left(D_{u}^{R}+D_{v}^{R}\right)\right]u_{re^{\prime }}\\ \;+[\Delta_{0}+\Lambda_{\beta }^{E}\left(a_{22}D_{u}^{E}+a_{11}D_{v}^{E}\right)+\Lambda_{\alpha }^{R}\left(a_{22}D_{u}^{R}+a_{11}D_{v}^{R}\right)\\ \;+{\left(\Lambda_{\beta }^{E}\right)}^{2}D_{u}^{E}D_{v}^{E}+\Lambda_{\beta }^{E}\Lambda_{\alpha }^{R}\left(D_{u}^{E}D_{v}^{R}+D_{u}^{R}D_{v}^{E}\right)\\ \;+{\left(\Lambda_{\alpha }^{R}\right)}^{2}D_{u}^{R}D_{v}^{R}]u_{re}=0. \end{array} \end{array}$$

Similarly, we get a second-order ODE about *v*_*re*_.

According to Lemma 2, a sufficient condition (H 2) for Turing instability caused by the Cartesian product network at (0, 0) is obtained. Of course, networks do not always cause instability.

### 2.2. The Hopf bifurcation of FHN network caused by delay

Suppose that (0, 0) in Equation (3) is stable, we next consider the effect of time delay on (0, 0). Adding time delay to the FHN network model (Equation 3), we have


(5) $$\begin{array}{l} \frac{du_{re}}{dt}=a_{11}u_{re}+a_{12}v_{re}\left(t-\tau \right)+f\left(u_{re}\right)+{\text{L}}_{u}u_{re},\\ \frac{dv_{re}}{dt}=a_{21}u_{re}+a_{22}v_{re}+{\text{L}}_{v}v_{re}. \end{array}$$

The Jacobian matrix of each node becomes


$$\begin{array}{r} J_{re}=\left(\begin{array}{cc} a_{11}+D_{u}^{R}\Lambda_{\alpha }^{R}+D_{u}^{E}\Lambda_{\beta }^{E} & 0\\ a_{21} & a_{22}+D_{v}^{R}\Lambda_{\alpha }^{R}+D_{v}^{E}\Lambda_{\beta }^{E} \end{array}\right)\\ +\left(\begin{array}{cc} 0 & a_{12}\\ 0 & 0 \end{array}\right){\text{e}}^{-\lambda_{re}\tau }≜J^{0}+J^{1}{\text{e}}^{-\lambda_{re}\tau }. \end{array}$$

Then the transcendental equation of the system (Equation 5) at (0, 0) is


$$\lambda_{re}^{2}+B_{1}\lambda_{re}+B_{2}+B_{3}{\text{e}}^{-\lambda_{re}\tau }=0,$$

where


$$\begin{array}{l} \begin{array}{l} B_{1}=-Tr_{0}-\left(D_{u}^{E}+D_{v}^{E}\right)\Lambda_{\beta }^{E}-\left(D_{u}^{R}+D_{v}^{R}\right)\Lambda_{\alpha }^{R},\\ B_{2}=\left(D_{v}^{E}\Lambda_{\beta }^{E}+D_{v}^{R}\Lambda_{\alpha }^{R}+a_{22}\right)\left(D_{u}^{E}\Lambda_{\beta }^{E}+D_{u}^{R}\Lambda_{\alpha }^{R}+a_{11}\right),\\ B_{3}=-a_{12}a_{21}. \end{array} \end{array}$$

Suppose *ιω* (ω > 0) be a root of the transcendental equation. And substituting *ιω* into the above equation, we can obtain


$$-\omega^{2}+B_{2}+B_{3}cos\left(\omega \tau \right)+\iota \left(B_{1}\omega -B_{3}sin\left(\omega \tau \right)\right)=0.$$

Comparing the coefficients, we have


$$\{\begin{array}{l} B_{3}cos\left(\omega \tau \right)=\omega^{2}-B_{2},\\ B_{3}sin\left(\omega \tau \right)=B_{1}\omega , \end{array}$$

then we obtain


$$\omega^{4}+\left(B_{1}^{2}-2B_{2}\right)\omega^{2}+B_{2}^{2}-B_{3}^{2}=0.$$

Let $x=\omega^{2},\;p=B_{1}^{2}-2B_{2},\;q=B_{2}^{2}-B_{3}^{2}$, then the equation becomes


(6) $$\begin{array}{l} x^{2}+px+q=0. \end{array}$$

**Lemma 3** Assume that (0, 0) in Equation (3) is stable. If 4*q* < 0 ≤ *p*^2^ and *p*>0, then the real parts of all roots of the transcendental equation are less than 0 for τ ∈ [0, τ_0_) and $\frac{dRe\lambda_{re}\left(\tau_{0}\right)}{d\tau }\neq 0$.

**Proof** The Equation (6) has only one positive root when 4*q* < 0 ≤ *p*^2^ and *p* > 0, denoted by *x*_0_. Hence, $\iota \omega_{0}=\iota \sqrt{x_{0}}$ is a purely imaginary root of the transcendental equation. Let


$$\tau_{0}^{j}\left(\Lambda_{\alpha }^{R},\Lambda_{\beta }^{E}\right)=\frac{1}{\omega_{0}}\arccos\frac{\omega_{0}^{2}-B_{2}}{B_{3}}+2\pi j,\;j=0,1,2,\cdot \cdot \cdot .$$

Define


(7) $$\begin{array}{l} \tau_{0}=\min_{\begin{array}{c} j\geq 1 \end{array}}\tau_{0}^{j}\left(\Lambda_{\alpha }^{R},\Lambda_{\beta }^{E}\right). \end{array}$$

Then again, τ_0_ is the minimum value of $\tau_{0}^{j}$, so the real parts of all roots of the transcendental equation are less than 0 for τ ∈ [0, τ_0_).

Next we prove the transversal condition. Let


$$\lambda_{re}\left(\tau \right)=\eta \left(\tau \right)+\iota \omega \left(\tau \right)$$

be the root of transcendental equation, then η(τ_0_) = 0, ω(τ_0_) = ω_0_. By taking the derivative with respect to τ in the transcendental equation, we can get


$$\frac{d\lambda_{re}\left(\tau \right)}{d\tau }=\frac{B_{3}\lambda_{re}{\text{e}}^{-\lambda_{re}\tau }}{2\lambda_{re}+B_{1}-B_{3}\tau {\text{e}}^{-\lambda_{re}\tau }}.$$

Substituting ω_0_, τ_0_ into the above equation, we can obtain


$$\frac{dRe\lambda_{re}\left(\tau_{0}\right)}{d\tau }=\frac{\omega_{0}^{2}}{\Theta }\left(2\omega_{0}^{2}+p\right),$$

where


$$\Theta ={\left(-\omega_{0}^{2}\tau_{0}+B_{2}\tau_{0}+B_{1}\right)}^{2}+{\left(B_{1}\omega_{0}\tau_{0}+2\omega_{0}\right)}^{2}.$$

So


$$\frac{dRe\lambda_{re}\left(\tau_{0}\right)}{d\tau }\neq 0.$$

According to the above analysis, the system (Equation 5) will occur Hopf bifurcation at τ = τ_0_ when Lemma 3 holds. Next, we discuss the properties of periodic solutions. The idea is: firstly, the system is written in the form of abstract ODE by using the infinitesimal generators theorem; then, A two-dimensional ODE that is the restriction to its center manifold is obtained by using the spectral decomposition theorem and the central manifold theorem of infinite dimensional systems; finally, the Hassard method is applied to determine the bifurcation attributes' parameters. The delay τ is taken as the control parameter, and let τ = τ_0_ + ò, *t* = τς. For convenience, we'll still use *t* to stand for ς. Setting $ℵ\left(t\right)={\left(u_{re}\left(t\right),v_{re}\left(t\right)\right)}^{T}$ be the solution of system (Equation 5) and define ℵ_*t*_(θ) = ℵ(*t* + θ), θ ∈ [−1, 0].

The system (Equation 5) is transformed into the following functional equation,


(8) $$\begin{array}{l} \dot{{ℵ}_{t}}={\text{AE}}_{ò}{ℵ}_{t}+F_{ò}\left({ℵ}_{t}\right), \end{array}$$

where linear operator ${\text{AE}}_{ó}:C\left(\left[-1,0\right],{\mathbb{R}}^{2}\right)≜C\to {\mathbb{R}}^{2}$,


$${\text{AE}}_{ó}\phi =\left(\tau_{0}+ò\right)J^{0}\phi \left(0\right)+\left(\tau_{0}+ò\right)J^{1}\phi \left(-1\right);$$

nonlinear operator $F_{ò}:C\to {\mathbb{R}}^{2}$,


$$\begin{array}{l} \;F_{ò}\left(\phi \right)=\left(\tau_{0}+ò\right)\left(\begin{array}{c} -u^{*}\phi_{1}{\left(0\right)}^{2}-\frac{\phi_{1}{\left(0\right)}^{3}}{3}\\ 0 \end{array}\right),\\ \text{where }\phi \left(\theta \right)={\left(\phi_{1}\left(\theta \right),\phi_{2}\left(\theta \right)\right)}^{T}. \end{array}$$

From Riesz representation theorem, there exists matrix η(θ, ò) of bounded variation functions satisfying


$${\text{AE}}_{ó}\phi =\int_{-1}^{0}\phi \left(\theta \right)\text{d}\eta \left(\theta ,ò\right),\text{ where }\phi \in C.$$

Let


$$\eta \left(\theta ,ò\right)=\left(\tau_{0}+ò\right)J^{0}\delta \left(\theta \right)+\left(\tau_{0}+ò\right)J^{1}\delta \left(\theta +1\right),$$

where δ(·) denotes Dirac function. According to the infinitesimal generators theorem, the abstract differential equation can be obtained from Equation (8)


(9) $$\begin{array}{l} \dot{{ℵ}_{t}}=A_{ò}{ℵ}_{t}+R_{ò}\left({ℵ}_{t}\right), \end{array}$$

where


$$\begin{array}{l} \begin{array}{l} A_{ò}\phi \left(\theta \right)=\{\begin{array}{l} \frac{\text{d}\phi \left(\theta \right)}{\text{d}\theta },\;\theta \in \left[-1,0\right),\\ \int_{-1}^{0}{\text{d}}_{\sigma }\eta \left(ò,\sigma \right)\phi \left(\sigma \right),\;\theta =0; \end{array}\\ R_{ò}\left(\phi \left(\theta \right)\right)=\{\begin{array}{l} 0,\;\theta \in \left[-1,0\right),\\ F_{ò}\left(\phi \right),\;\theta =0. \end{array} \end{array} \end{array}$$

In the following, we will discuss ODE (Equation 9) by using formal adjoint theorem, center manifold theorem and normal form theory.

Let $A_{ò}^{*}$ be the conjugate operator of *A*_ò_. According to the formal adjoint theorem, there is


$$A_{ò}^{*}\psi \left(s\right)=\{\begin{array}{l} -\frac{\text{d}\psi \left(s\right)}{\text{d}s},\;s\in \left(0,1\right],\\ \int_{-1}^{0}\text{d}\eta^{T}\left(\sigma ,0\right)\psi \left(-\sigma \right),\;s=0. \end{array}$$

Define product


$$\langle \psi \left(s\right),\phi \left(\theta \right)\rangle ={\bar{\psi }}^{T}\left(0\right)\phi \left(0\right)-\int_{\theta =-1}^{0}\int_{\xi =0}^{\theta }{\bar{\psi }}^{T}\left(\xi -\theta \right)\text{d}\eta \left(\theta \right)\phi \left(\xi \right)\text{d}\xi ,$$

which satisfies $\langle \psi ,A_{ò}\phi \rangle =\langle A_{ò}^{*}\psi ,\phi \rangle$ and η(θ) = η(θ, 0). From the previous discussion, we can obtain that ±*ιω*_0_τ_0_ are the eigenvalues of *A*_0_, $A_{0}^{*}$.

**Lemma 4** Let $q\left(\theta \right)={\left(1,q_{2}\right)}^{T}{\text{e}}^{\iota \omega_{0}\tau_{0}\theta }$ be the eigenvector of *A*_0_ corresponding to *ιω*_0_τ_0_, and $q^{*}\left(s\right)=\kappa {\left(q_{1}^{*},1\right)}^{T}{\text{e}}^{\iota \omega_{0}\tau_{0}s}$ be the eigenvector of $A_{0}^{*}$ corresponding to −*ιω*_0_τ_0_. And let 〈q^*^(*s*), *q*(θ)〉 = 1, then we can choose


$$\begin{array}{l} q_{2}=\frac{a_{21}}{\iota \omega_{0}-a_{22}},\;q_{1}^{*}=\frac{-a_{21}}{\iota \omega_{0}+a_{11}},\\ \kappa =\frac{1}{q_{1}^{*}+\bar{q_{2}}+\tau_{0}a_{12}q_{1}^{*}\bar{q_{2}}{\text{e}}^{\iota \omega_{0}\tau_{0}}}. \end{array}$$

**Proof** From the hypothesis, we have


$$A_{0}\left(\begin{array}{c} 1\\ q_{2} \end{array}\right)=\iota \omega_{0}\tau_{0}\left(\begin{array}{c} 1\\ q_{2} \end{array}\right),\;A_{0}^{*}\left(\begin{array}{c} q_{1}^{*}\\ 1 \end{array}\right)=-\iota \omega_{0}\tau_{0}\left(\begin{array}{c} q_{1}^{*}\\ 1 \end{array}\right).$$

Then


$$q_{2}=\frac{a_{21}}{\iota \omega_{0}-a_{22}},\;q_{1}^{*}=\frac{-a_{21}}{\iota \omega_{0}+a_{11}}.$$

Next, we calculate the expression of κ. According to the bilinear inner product formula, we have


$$\begin{array}{l} \begin{array}{l} \langle q^{*}\left(s\right),q\left(\theta \right)\rangle ={\bar{q^{*}}}^{T}\left(0\right)q\left(0\right)-\int_{-1}^{0}\int_{0}^{\theta }{\bar{q^{*}}}^{T}\left(\xi -\theta \right)\text{d}\eta \left(\theta \right)q\left(\xi \right)\text{d}\xi \\ \;=\bar{\kappa }\left(\bar{q_{1}^{*}},1\right){\left(1,q_{2}\right)}^{T}\\ \;-\int_{-1}^{0}\int_{0}^{\theta }\bar{\kappa }\left(\bar{q_{1}^{*}},1\right){\text{e}}^{-\iota \omega_{0}\tau_{0}\left(\xi -\theta \right)}\text{d}\eta \left(\theta \right){\left(1,q_{2}\right)}^{T}{\text{e}}^{\iota \omega_{0}\tau_{0}\xi }\text{d}\xi \\ \;=\bar{\kappa }\left[\bar{q_{1}^{*}}+q_{2}-\left(\bar{q_{1}^{*}},1\right)\int_{-1}^{0}\theta {\text{e}}^{\iota \omega_{0}\tau_{0}\theta }\text{d}\eta \left(\theta \right){\left(1,q_{2}\right)}^{T}\right]\\ \;=\bar{\kappa }\left[\bar{q_{1}^{*}}+q_{2}+\left(\bar{q_{1}^{*}},1\right)\tau_{0}J^{1}{\text{e}}^{-\iota \omega_{0}\tau_{0}}{\left(1,q_{2}\right)}^{T}\right]\\ \;=\bar{\kappa }\left[\bar{q_{1}^{*}}+q_{2}+\tau_{0}a_{12}\bar{q_{1}^{*}}q_{2}{\text{e}}^{-\iota \omega_{0}\tau_{0}}\right]. \end{array} \end{array}$$

To make 〈q^*^(*s*), *q*(θ)〉 = 1, we take


$$\bar{\kappa }=\frac{1}{\bar{q_{1}^{*}}+q_{2}+\tau_{0}a_{12}\bar{q_{1}^{*}}q_{2}{\text{e}}^{-\iota \omega_{0}\tau_{0}}}.$$

The center manifold Ω_0_ of Equation (8) is locally invariant when ò = 0. To achieve spectral decomposition, we build the local coordinates *z* and $\bar{z}$ on the center manifold Ω_0_. Let *U*_*t*_ = *U*_*t*_(θ) be the solution of the system (Equation 8) when ò = 0, then


$$z\left(t\right)=\langle q^{*},U_{t}\rangle .$$

And let


(10) $$\begin{array}{l} W\left(t,\theta \right)=U_{t}\left(\theta \right)-z\left(t\right)q\left(\theta \right)-\bar{z}\left(t\right)\bar{q}\left(\theta \right). \end{array}$$

$W\left(t,\theta \right)=W\left(z,\bar{z},\theta \right)$ on the center manifold Ω_0_, so $W\left(z,\bar{z},\theta \right)$ can be expanded as


(11) $$\begin{array}{l} W\left(z,\bar{z},\theta \right)=W_{20}\left(\theta \right)\frac{z^{2}}{2}+W_{11}\left(\theta \right)z\bar{z}+W_{02}\left(\theta \right)\frac{{\bar{z}}^{2}}{2}+\cdot \cdot \cdot . \end{array}$$

*W* is real when *U*_*t*_ is real. Therefore, in this case, let's just look at the real solution. Obviously, there is


$$\langle q^{*},W\rangle =0.$$

Because of the existence of the center manifold, it is possible to transform the functional differential equation (Equation 8) into simple complex variable ODE on Ω. When ò = 0, there is


(12) $$\begin{array}{l} \begin{array}{l} \dot{z}\left(t\right)=\langle q^{*},\dot{U_{t}}\rangle \\ \;=\iota \omega_{0}\tau_{0}z\left(t\right)+{\bar{q^{*}}}^{T}\left(0\right)F_{0}\left(W\left(z,\bar{z},\theta \right)+zq\left(\theta \right)+\bar{z}\bar{q}\left(\theta \right)\right)\\ \;≜\iota \omega_{0}\tau_{0}z\left(t\right)+g\left(z,\bar{z}\right)\left(t\right). \end{array} \end{array}$$

And since *F*_ò_(ϕ) is at least quadratic with respect to ϕ, we can write


(13) $$\begin{array}{l} g\left(z,\bar{z}\right)=g_{20}\frac{z^{2}}{2}+g_{11}z\bar{z}+g_{02}\frac{{\bar{z}}^{2}}{2}+g_{21}\frac{z^{2}\bar{z}}{2}+\cdot \cdot \cdot . \end{array}$$

By combining Equations (10), (11), we can obtain


$$\begin{array}{l} \begin{array}{l} U_{t}\left(\theta \right)=W\left(t,\theta \right)+z\left(t\right)q\left(\theta \right)+\bar{z}\left(t\right)\bar{q}\left(\theta \right)\\ \;={\left(1,q_{2}\right)}^{T}{\text{e}}^{\iota \omega_{0}\tau_{0}\theta }z+{\left(1,{\bar{q}}_{2}\right)}^{T}{\text{e}}^{-\iota \omega_{0}\tau_{0}\theta }\bar{z}+W_{20}\left(\theta \right)\frac{z^{2}}{2}\\ \;+W_{11}\left(\theta \right)z\bar{z}+W_{02}\left(\theta \right)\frac{{\bar{z}}^{2}}{2}+\cdot \cdot \cdot . \end{array} \end{array}$$

Substituting the above equation into (13), it can be obtained


$$\begin{array}{l} \begin{array}{l} g\left(z,\bar{z}\right)={\bar{q^{*}}}^{T}\left(0\right)F_{0}\left(U_{t}\right)=\tau_{0}\bar{\kappa }\left(\bar{q_{1}^{*}},1\right)\left(\begin{array}{c} -u^{*}\phi_{1}{\left(0\right)}^{2}-\frac{\phi_{1}{\left(0\right)}^{3}}{3}\\ 0 \end{array}\right)\\ =-\tau_{0}\bar{\kappa }\bar{q_{1}^{*}}[u^{*}(z+\bar{z}+W_{20}^{\left(1\right)}\left(0\right)\frac{z^{2}}{2}+W_{11}^{\left(1\right)}\left(0\right)z\bar{z}+W_{02}^{\left(1\right)}\left(0\right)\frac{{\bar{z}}^{2}}{2}+\cdot \cdot \cdot {)}^{2}\\ +\frac{1}{3}(z+\bar{z}+W_{20}^{\left(1\right)}\left(0\right)\frac{z^{2}}{2}+W_{11}^{\left(1\right)}\left(0\right)z\bar{z}+W_{02}^{\left(1\right)}\left(0\right)\frac{{\bar{z}}^{2}}{2}+\cdot \cdot \cdot {)}^{3}]\\ =-\tau_{0}\bar{\kappa }\bar{q_{1}^{*}}[u^{*}{\bar{z}}^{2}+2u^{*}z\bar{z}+u^{*}z^{2}\\ +\left(2u^{*}W_{11}^{\left(1\right)}\left(0\right)+u^{*}W_{20}^{\left(1\right)}\left(0\right)+1\right)z^{2}\bar{z}+\cdot \cdot \cdot ]. \end{array} \end{array}$$

Obviously, there are


(14) $$\begin{array}{l} g_{02}=g_{11}=g_{20}=-2\tau_{0}\bar{\kappa }\bar{q_{1}^{*}}u^{*},\\ g_{21}=-2\tau_{0}\bar{\kappa }\bar{q_{1}^{*}}\left(2u^{*}W_{11}^{\left(1\right)}\left(0\right)+u^{*}W_{20}^{\left(1\right)}\left(0\right)+1\right). \end{array}$$

Observing the above equation, we can see that if we want to calculate *g*_21_, we must first calculate *W*_20_(θ) and *W*_11_(θ). Next, we determine the exact expression for *W*_20_(θ), *W*_11_(θ).

According to Equations (9), (10), and (12), we have


(15) $$\begin{array}{l} \dot{W}=\dot{U_{t}}-\dot{z}q-\dot{\bar{z}}\bar{q}\\ \;=\{\begin{array}{ll} A_{0}W-gq\left(\theta \right)-\bar{g}\bar{q}\left(\theta \right), & -1\leq \theta <0\\ A_{0}W-gq\left(\theta \right)-\bar{g}\bar{q}\left(\theta \right)+F_{0}, & \theta =0 \end{array}\\ \;≜A_{0}W+M\left(z,\bar{z},\theta \right), \end{array}$$

where


(16) $$\begin{array}{l} M\left(z,\bar{z},\theta \right)=M_{20}\left(\theta \right)\frac{z^{2}}{2}+M_{11}\left(\theta \right)z\bar{z}+M_{02}\left(\theta \right)\frac{{\bar{z}}^{2}}{2}+\cdot \cdot \cdot . \end{array}$$

Combining Equations (11), (15), and (16), $\dot{W}$ can be expressed as


(17) $$\begin{array}{l} \dot{W}=A_{0}\left[W_{20}\left(\theta \right)\frac{z^{2}}{2}+W_{11}\left(\theta \right)z\bar{z}+W_{02}\left(\theta \right)\frac{{\bar{z}}^{2}}{2}+\cdot \cdot \cdot \right]\\ \;+M_{20}\left(\theta \right)\frac{z^{2}}{2}+M_{11}\left(\theta \right)z\bar{z}+M_{02}\left(\theta \right)\frac{{\bar{z}}^{2}}{2}+\cdot \cdot \cdot \\ \;=\left[A_{0}W_{20}\left(\theta \right)+M_{20}\left(\theta \right)\right]\frac{z^{2}}{2}+\left[A_{0}W_{11}\left(\theta \right)+M_{11}\left(\theta \right)\right]z\bar{z}\\ \;+\left[A_{0}W_{02}\left(\theta \right)+M_{02}\left(\theta \right)\right]\frac{{\bar{z}}^{2}}{2}+\cdot \cdot \cdot . \end{array}$$

On the other hand, combining Equations (11), (12), we know that on the center manifold Ω_0_ near the origin, $\dot{W}$ can also be expressed as


(18) $$\begin{array}{l} \dot{W}=\left(W_{20}z+W_{11}\bar{z}+\cdot \cdot \cdot \right)\left[\iota \omega_{0}\tau_{0}z+g\left(z,\bar{z}\right)\right]\\ \;+\left(W_{11}z+W_{02}\bar{z}+\cdot \cdot \cdot \right)\left[-\iota \omega_{0}\tau_{0}\bar{z}+\bar{g}\left(z,\bar{z}\right)\right]\\ \;=2\iota \omega_{0}\tau_{0}\left(W_{20}\frac{z^{2}}{2}+W_{02}\frac{{\bar{z}}^{2}}{2}+\cdot \cdot \cdot \right). \end{array}$$

Comparing the coefficients of *z*^2^ and $z\bar{z}$ in Equations (17), (18), the relationship between *W*_*ij*_(θ) and *M*_*ij*_(θ) can be obtained


(19) $$\begin{array}{l} \left(2\iota \omega_{0}\tau_{0}𝕀-A_{0}\right)W_{20}\left(\theta \right)=M_{20}\left(\theta \right),\;-A_{0}W_{11}\left(\theta \right)=M_{11}\left(\theta \right). \end{array}$$

Next, we will determine *W*_11_(θ) and *W*_20_(θ) according to the relationship between $g\left(z,\bar{z}\right)$ and $M\left(z,\bar{z},\theta \right)$.

When −1 ≤ θ < 0, combining Equations (15), (16), it is clear that


(20) $$\begin{array}{l} \begin{array}{l} M_{20}\left(\theta \right)=-g_{20}q\left(\theta \right)-{\bar{g}}_{02}\bar{q}\left(\theta \right),\\ M_{11}\left(\theta \right)=-g_{11}q\left(\theta \right)-{\bar{g}}_{11}\bar{q}\left(\theta \right). \end{array} \end{array}$$

Combining Equations (19), (20) and the definition of *A*_ò_, we get


(21) $$\begin{array}{l} \frac{dW_{20}}{d\theta }=2\iota \omega_{0}\tau_{0}W_{20}\left(\theta \right)+g_{20}q\left(\theta \right)+{\bar{g}}_{02}\bar{q}\left(\theta \right),\\ \frac{{dW}_{11}}{d\theta }=g_{11}q\left(\theta \right)+{\bar{g}}_{11}\bar{q}\left(\theta \right). \end{array}$$

By substituting $q\left(\theta \right)={\left(1,q_{2}\right)}^{T}{\text{e}}^{\iota \omega_{0}\tau_{0}\theta }$ into the above equation, it can be obtained by the constant variation method


(22) $$\begin{array}{l} W_{20}\left(\theta \right)=\frac{\iota g_{20}}{\omega_{0}\tau_{0}}q\left(0\right){\text{e}}^{\iota \omega_{0}\tau_{0}\theta }+\frac{\iota {\bar{g}}_{02}}{3\omega_{0}\tau_{0}}\bar{q}\left(0\right){\text{e}}^{-\iota \omega_{0}\tau_{0}\theta }+\ell_{1}{\text{e}}^{2\iota \omega_{0}\tau_{0}\theta },\\ W_{11}\left(\theta \right)=-\frac{\iota g_{11}}{\omega_{0}\tau_{0}}q\left(0\right){\text{e}}^{\iota \omega_{0}\tau_{0}\theta }+\frac{\iota {\bar{g}}_{11}}{\omega_{0}\tau_{0}}\bar{q}\left(0\right){\text{e}}^{-\iota \omega_{0}\tau_{0}\theta }+\ell_{2}, \end{array}$$

Where $\ell_{1}={\left(\ell_{1}^{1},\ell_{1}^{2}\right)}^{T},\;\ell_{2}={\left(\ell_{2}^{1},\ell_{2}^{2}\right)}^{T}$ are two dimensional constant vectors. Next, let's figure out what the values of ℓ_1_ and ℓ_2_ are.

According to Equation (19) and the definition of *A*_0_, when θ = 0, there is


(23) $$\begin{array}{l} \begin{array}{l} \int_{-1}^{0}\text{d}\eta \left(\theta \right)W_{20}\left(\theta \right)=2\iota \omega_{0}\tau_{0}W_{20}\left(0\right)-M_{20}\left(0\right),\\ \int_{-1}^{0}\text{d}\eta \left(\theta \right)W_{11}\left(\theta \right)=-M_{11}\left(0\right). \end{array} \end{array}$$

When θ = 0, combining Equations (15), (16), it is clear that


(24) $$\begin{array}{l} M_{20}\left(0\right)=-g_{20}q\left(0\right)-{\bar{g}}_{02}\bar{q}\left(0\right)+2\tau_{0}\left(\begin{array}{c} -u^{*}\\ 0 \end{array}\right),\\ M_{11}\left(0\right)=-g_{11}q\left(0\right)-{\bar{g}}_{11}\bar{q}\left(0\right)+2\tau_{0}\left(\begin{array}{c} -u^{*}\\ 0 \end{array}\right). \end{array}$$

Since *q*(0) is the eigenvector of *A*_0_ corresponding to *ιω*_0_τ_0_, we can obtain


(25) $$\begin{array}{r} \left(\iota \omega_{0}\tau_{0}𝕀-\int_{-1}^{0}{\text{e}}^{\iota \omega_{0}\tau_{0}\theta }\text{d}\eta \left(\theta \right)\right)q\left(0\right)=0,\\ \left(-\iota \omega_{0}\tau_{0}𝕀-\int_{-1}^{0}{\text{e}}^{-\iota \omega_{0}\tau_{0}\theta }\text{d}\eta \left(\theta \right)\right)\bar{q}\left(0\right)=0. \end{array}$$

Substituting Equations (22), (24), and (25) into Equation (23), we obtain


(26) $$\begin{array}{l} \left(2\iota \omega_{0}\tau_{0}𝕀-\int_{-1}^{0}{\text{e}}^{2\iota \omega_{0}\tau_{0}\theta }\text{d}\eta \left(\theta \right)\right)\ell_{1}=2\tau_{0}\left(\begin{array}{c} -u^{*}\\ 0 \end{array}\right),\\ \int_{-1}^{0}\text{d}\eta \left(\theta \right)\ell_{2}=-2\tau_{0}\left(\begin{array}{c} -u^{*}\\ 0 \end{array}\right). \end{array}$$

When ò = 0, there are


$$\begin{array}{l} \left(\begin{array}{cc} 2\iota \omega_{0}-a_{11}-D_{u}^{R}\Lambda_{\alpha }^{R}-D_{u}^{E}\Lambda_{\beta }^{E} & -a_{12}{\text{e}}^{-2\iota \omega_{0}\tau_{0}}\\ -a_{21} & 2\iota \omega_{0}-a_{22}-D_{v}^{R}\Lambda_{\alpha }^{R}-D_{v}^{E}\Lambda_{\beta }^{E} \end{array}\right)\ell_{1}\\ =2\left(\begin{array}{c} -u^{*}\\ 0 \end{array}\right),\\ \left(\begin{array}{cc} -a_{11}-D_{u}^{R}\Lambda_{\alpha }^{R}-D_{u}^{E}\Lambda_{\beta }^{E} & -a_{12}\\ -a_{21} & -a_{22}-D_{v}^{R}\Lambda_{\alpha }^{R}-D_{v}^{E}\Lambda_{\beta }^{E} \end{array}\right)\ell_{2}=2\left(\begin{array}{c} -u^{*}\\ 0 \end{array}\right), \end{array}$$

that is,


$$\begin{array}{l} \ell_{1}=-2{\left(\begin{array}{cc} 2\iota \omega_{0}-a_{11}-D_{u}^{R}\Lambda_{\alpha }^{R}-D_{u}^{E}\Lambda_{\beta }^{E} & -a_{12}{\text{e}}^{-2\iota \omega_{0}\tau_{0}}\\ -a_{21} & 2\iota \omega_{0}-a_{22}-D_{v}^{R}\Lambda_{\alpha }^{R}-D_{v}^{E}\Lambda_{\beta }^{E} \end{array}\right)}^{-1}\\ \;\times \left(\begin{array}{c} u^{*}\\ 0 \end{array}\right),\\ \ell_{2}=-2{\left(\begin{array}{cc} -a_{11}-D_{u}^{R}\Lambda_{\alpha }^{R}-D_{u}^{E}\Lambda_{\beta }^{E} & -a_{12}\\ -a_{21} & -a_{22}-D_{v}^{R}\Lambda_{\alpha }^{R}-D_{v}^{E}\Lambda_{\beta }^{E} \end{array}\right)}^{-1}\left(\begin{array}{c} u^{*}\\ 0 \end{array}\right). \end{array}$$

Substituting ℓ_1_, ℓ_2_ into Equation (22), we can find *W*_20_ and *W*_11_. To date, *g*_20_, *g*_21_, *g*_11_ and *g*_02_ are now all found, and the normal form Equation (12) that is the restriction to its center manifold is obtained. The key parameters μ_2_, *T*_2_ and Floquet exponent β_2_ that determine the properties of periodic solutions can be calculated by Hassard's method,


(27) $$\begin{array}{l} \{\begin{array}{l} c_{1}\left(0\right)=\frac{\iota }{2\omega_{0}\tau_{0}}\left(g_{11}g_{20}-2|g_{11}{|}^{2}-\frac{1}{3}|g_{02}{|}^{2}\right)+\frac{1}{2}g_{21},\\ \mu_{2}=-\frac{Re\left[c_{1}\left(0\right)\right]}{Re\left[\lambda^{\prime }\left(\tau_{0}\right)\right]},\\ \beta_{2}=2Re\left[c_{1}\left(0\right)\right],\\ T_{2}=-\frac{Im\left[c_{1}\left(0\right)\right]+\mu_{2}Im\left[\lambda^{\prime }\left(\tau_{0}\right)\right]}{\omega_{0}}. \end{array} \end{array}$$

**Theorem 2** Suppose that the conditions of Lemma 3 are satisfied, then

(i) If μ_2_ > 0(< 0), the periodic solution is a supercritical (subcritical) Hopf bifurcation.
(ii) If *T*_2_ > 0(< 0), the period of the periodic solution increases (decreases) as τ moves away from τ_0_.
(iii) If β_2_ > 0(< 0), the periodic solutions restricted on the center manifold are orbitally asymptotically unstable (stable).

## 3. Simulation

**Figure 1:**
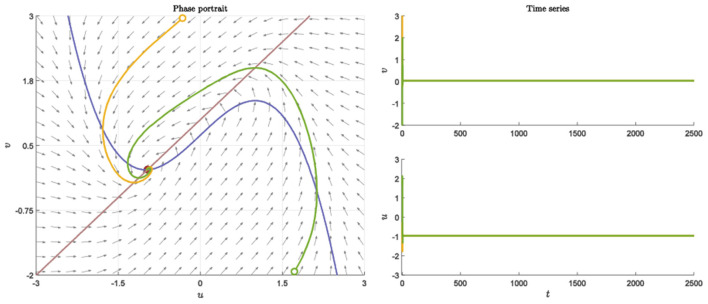
Nullcline, phase portraits with different initial value and time series when *a* = 1, *b* = 1, *c* = 2, *d* = 1, *I* = 0.7.

We perform simple simulations to verify the above theoretical results in this section. The topological properties of neural networks are very important to the dynamic behavior of neuronal clusters. In both *R* and *E*, we pick random networks with connection probability *p* and $D_{u}^{R}=D_{u}^{E}=D_{u},\;D_{v}^{R}=D_{v}^{E}=D_{v}$. And setting the parameters as *a* = 1, *b* = 1, *c* = 2, *d* = 1, *I* = 0.7, *n*_*r*_ = *n*_*e*_ = 20. Neurons still return to the resting state after receiving different stimuli (Figure 1).

In this case, condition (H 2) becomes


$$y=\Delta_{0}+\left(a_{22}D_{u}+a_{11}D_{v}\right)\left(\Lambda_{\alpha }^{R}+\Lambda_{\beta }^{E}\right)+D_{u}D_{v}{\left(\Lambda_{\alpha }^{R}+\Lambda_{\beta }^{E}\right)}^{2}<0.$$

**Figure 2:**
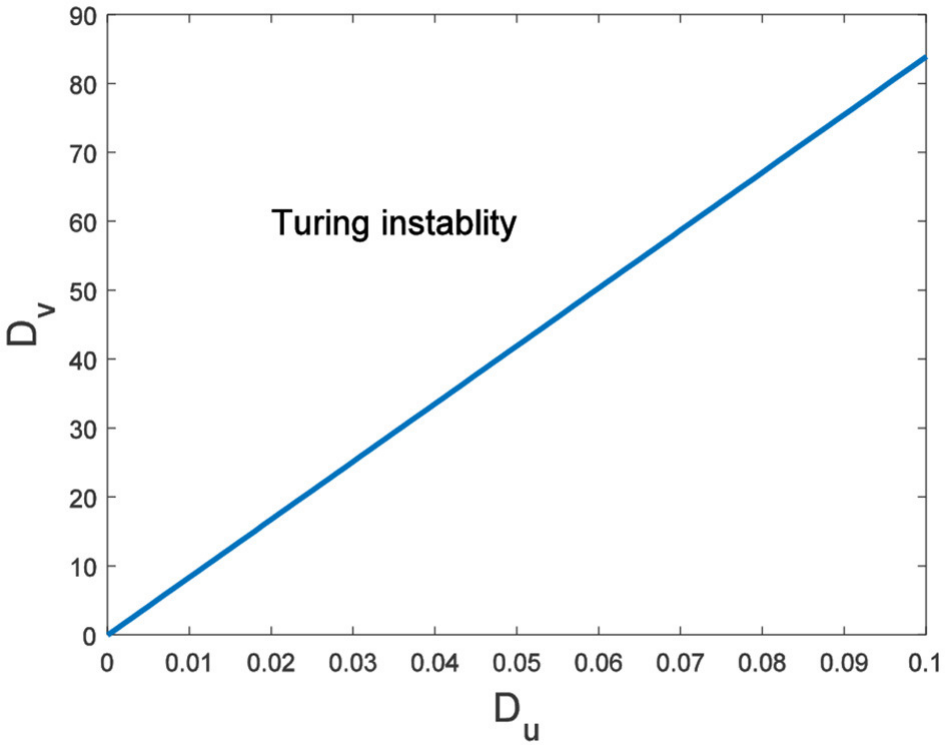
The range of Turing instability in the general diffusion system about *D*_*u*_ and *D*_*v*_.

**Figure 3:**
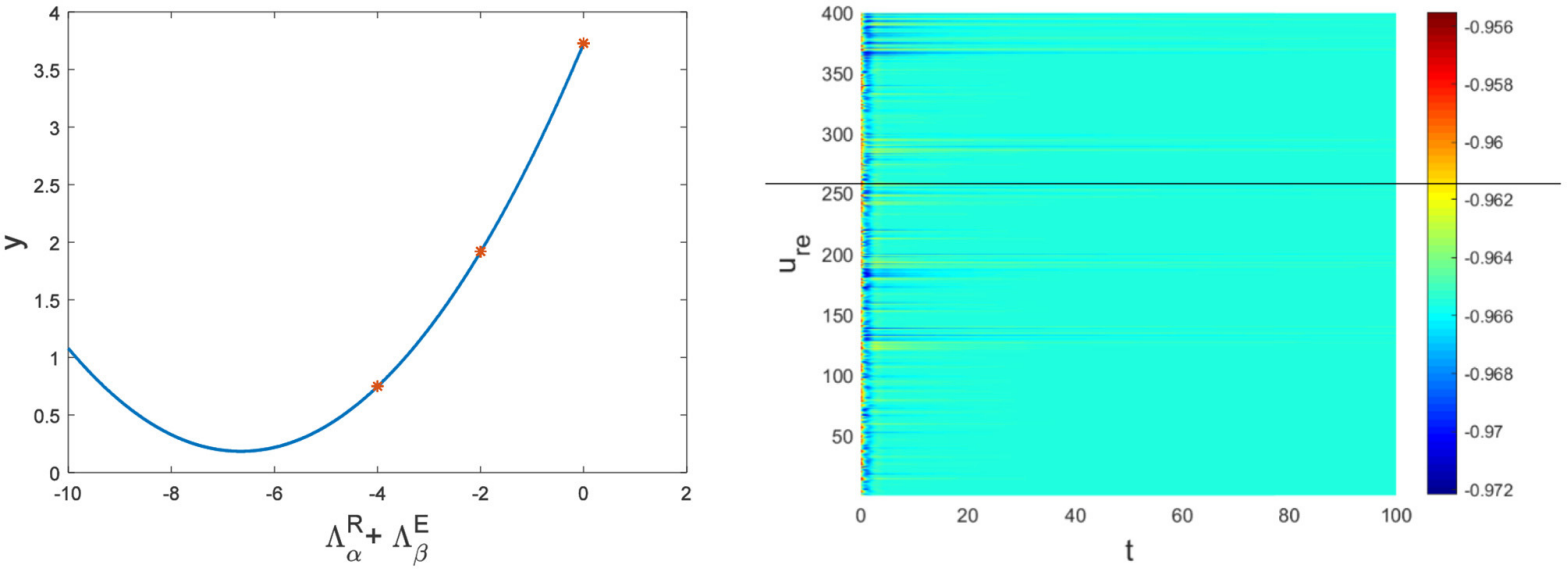
The relationship between $\Lambda_{\alpha }^{R}+\Lambda_{\beta }^{E}$ and *y* and the corresponding pattern when *a* = 1, *b* = 1, *c* = 2, *d* = 1, *I* = 0.7, *D*_*u*_ = 0.01, *D*_*v*_ = 8, *p* = 0.01. The red dots are the eigenvalues of the network Laplacian matrix.

**Figure 4:**
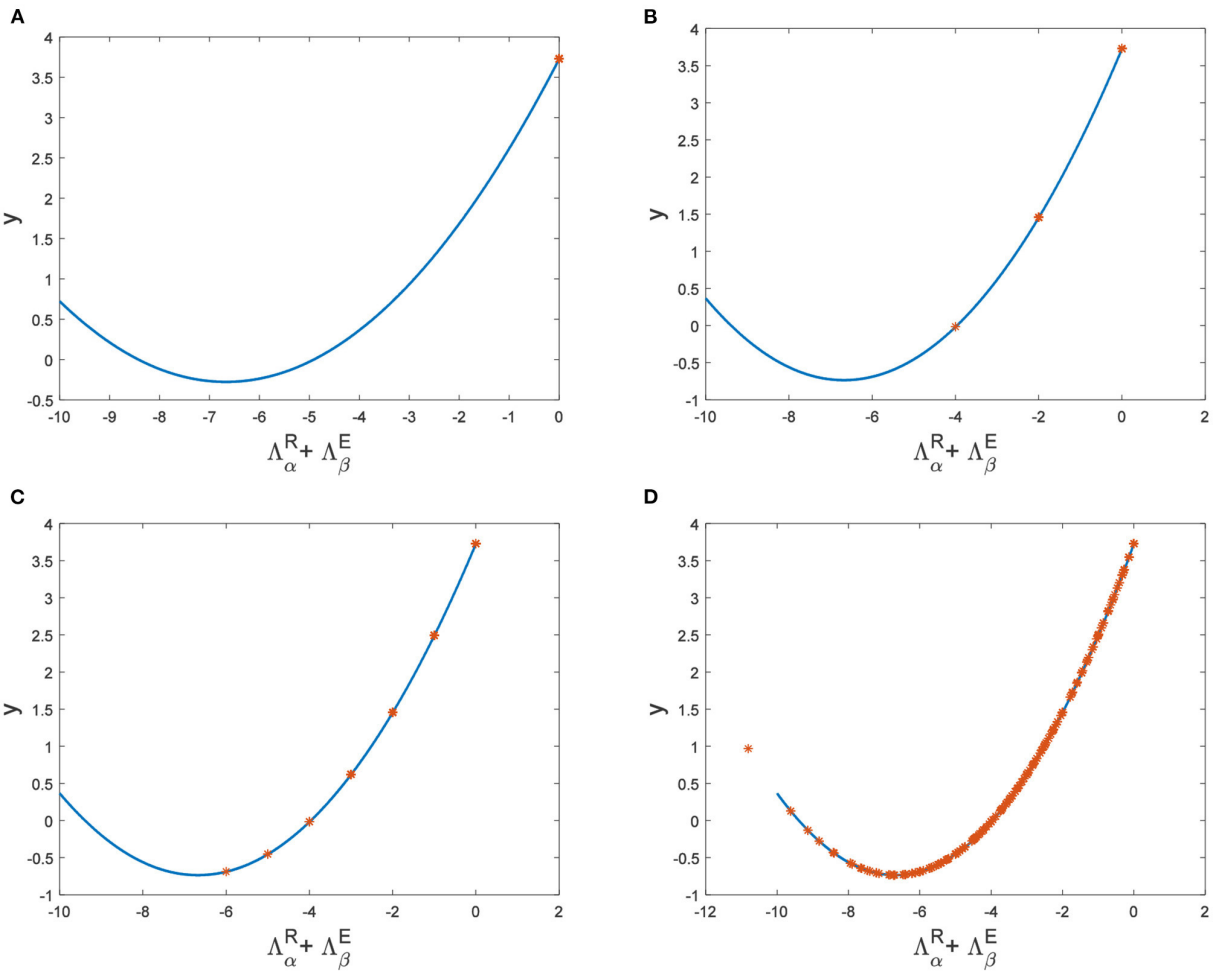
The relationship between $\Lambda_{\alpha }^{R}+\Lambda_{\beta }^{E}$ and *y* when *a* = 1, *b* = 1, *c* = 2, *d* = 1, *I* = 0.7, *D*_*u*_ = 0.01. **(A)** *D*_*v*_ = 9, *p* = 0.001. **(B)** *D*_*v*_ = 10, *p* = 0.006. **(C)** *D*_*v*_ = 10, *p* = 0.01. **(D)** *D*_*v*_ = 10, *p* = 0.1.

**Figure 5:**
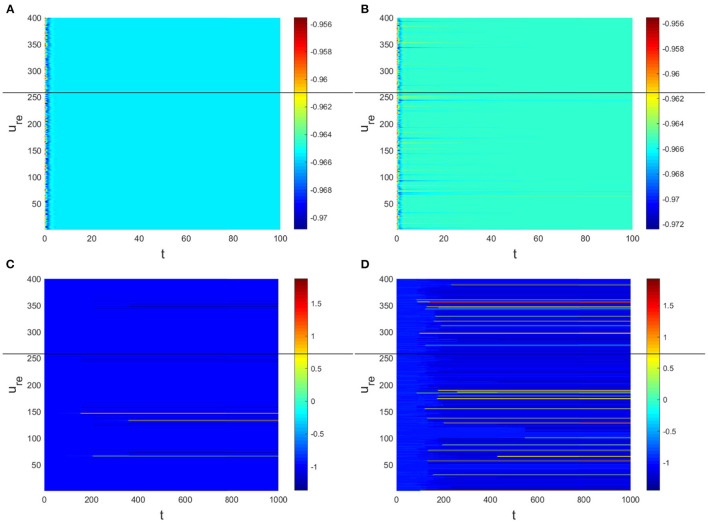
The corresponding Turing pattern in Figure 4. **(A)** *D*_*v*_ = 9, *p* = 0.001. **(B)** *D*_*v*_ = 10, *p* = 0.006. **(C)** *D*_*v*_ = 10, *p* = 0.01. **(D)** *D*_*v*_ = 10, *p* = 0.1.

Hence, Turing instability occurs in the general diffusion system when *a*_22_*D*_*u*_ + *a*_11_*D*_*v*_ > 0 and ${\left(a_{22}D_{u}+a_{11}D_{v}\right)}^{2}-4\Delta_{0}D_{u}D_{v}>0$. And the critical value is *D*_*v*_ = 8.3923 when *D*_*u*_ = 0.01 (Figure 2). Different dynamic behaviors [such as Hopf bifurcation (40) and chaos (41)] and various spatiotemporal patterns [such as irregular waves, target waves, traveling waves, and spiral waves (42, 43)] will appear when the system is subjected to different kinds and degrees of external stimulus. In the neural system, these spatiotemporal patterns are closely related to brain learning, memory, and information transmission. When the brain stores memory, the continuous firing rate of individual neurons shows a hierarchical change and the neurons show a strong temporal dynamic pattern and heterogeneity (33). Many factors contribute to the formation of short-term memory. Short-term memory does not form when the external stimulus is not sufficiently large (Figure 3). It is worth noting that neuronal activity is not only affected by external stimuli but also closely related to the interaction between nodes. The pattern remains flat when the external stimulus is large enough and the correlation degree of neurons is small. That is, short-term memory will not form (Figures 4A, B, 5A, B). When *p* increased to 0.01, neurons in the memory function areas fired, and the brain formed more vague memories (Figures 4C, D, 5C, D). Zheng et al. (37) found that neurons exhibit different pattern dynamics with the change of network connection probability *p* in the study of the HR model. This conclusion is also confirmed in the study of multilayer networks. Under the same degree of stimulation, if the number of neurons with the same functional attributes is different, the state of the neural network varies greatly (Figures 4B–D).

**Figure 6:**
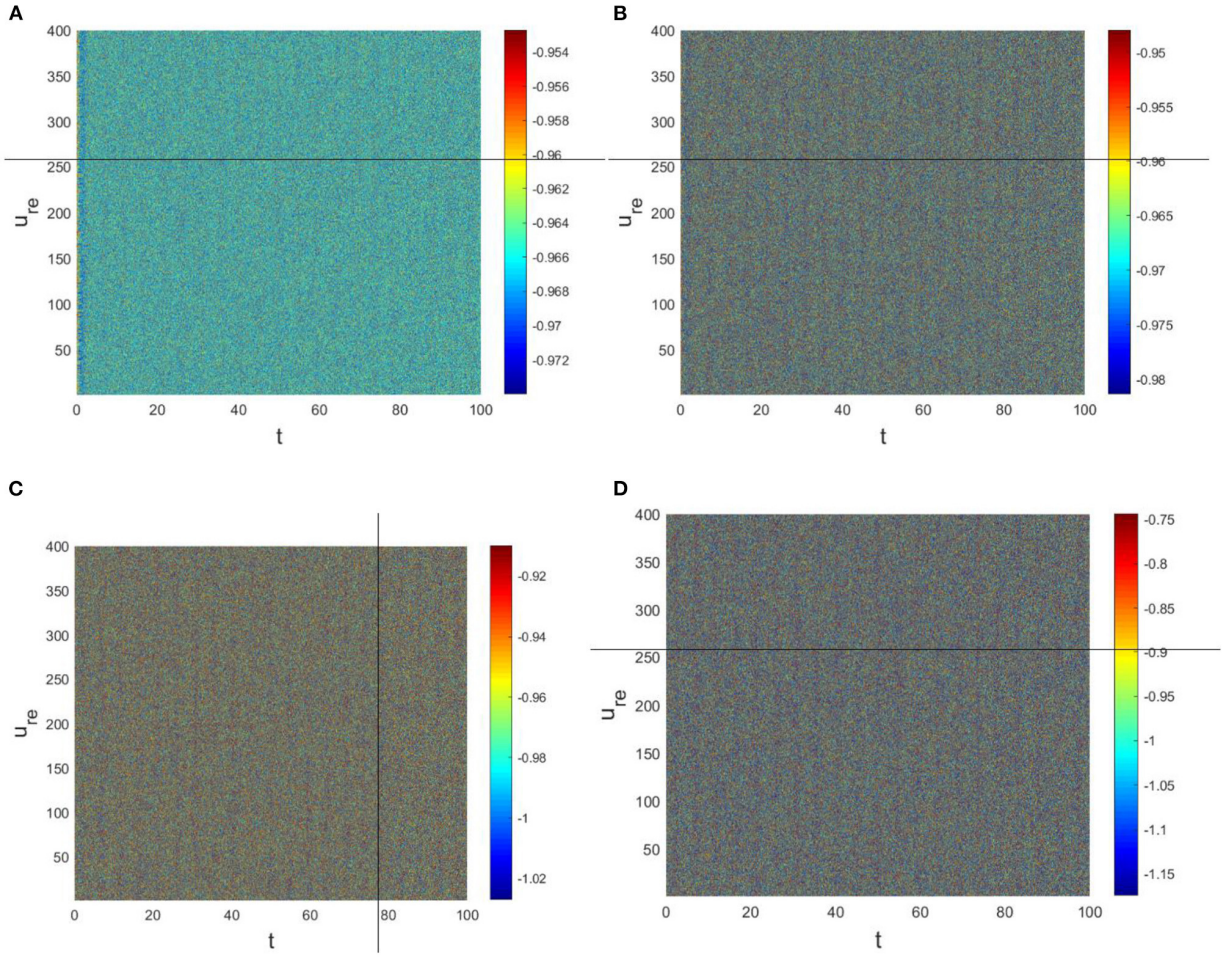
Pattern with *a* = 1, *b* = 1, *c* = 2, *d* = 1, *I* = 0.7, *D*_*u*_ = 0.01, *D*_*v*_ = 9, *p* = 0.001. **(A)** *np* = *O*(10^−7^). **(B)** *np* = *O*(10^−6^). **(C)** *np* = *O*(10^−4^). **(D)** *np* = *O*(10^−3^).

The physiological environment in which neurons work is always full of noise. From the above analysis, we can see that when *D*_*v*_ = 9, *p* = 0.001 is taken, the neurons are always in resting state (Figure 5A). To investigate the robustness of noise to the current results, we add Gaussian white noise to the multilayer FHN network model. The noise intensity *np* about *u* is used as the control parameter. We find that the system is robust when *np* < *O*(10^−5^); when *np* > *O*(10^−5^), the neurons are excited and the short-term memory is vague (Figure 6).

**Figure 7:**
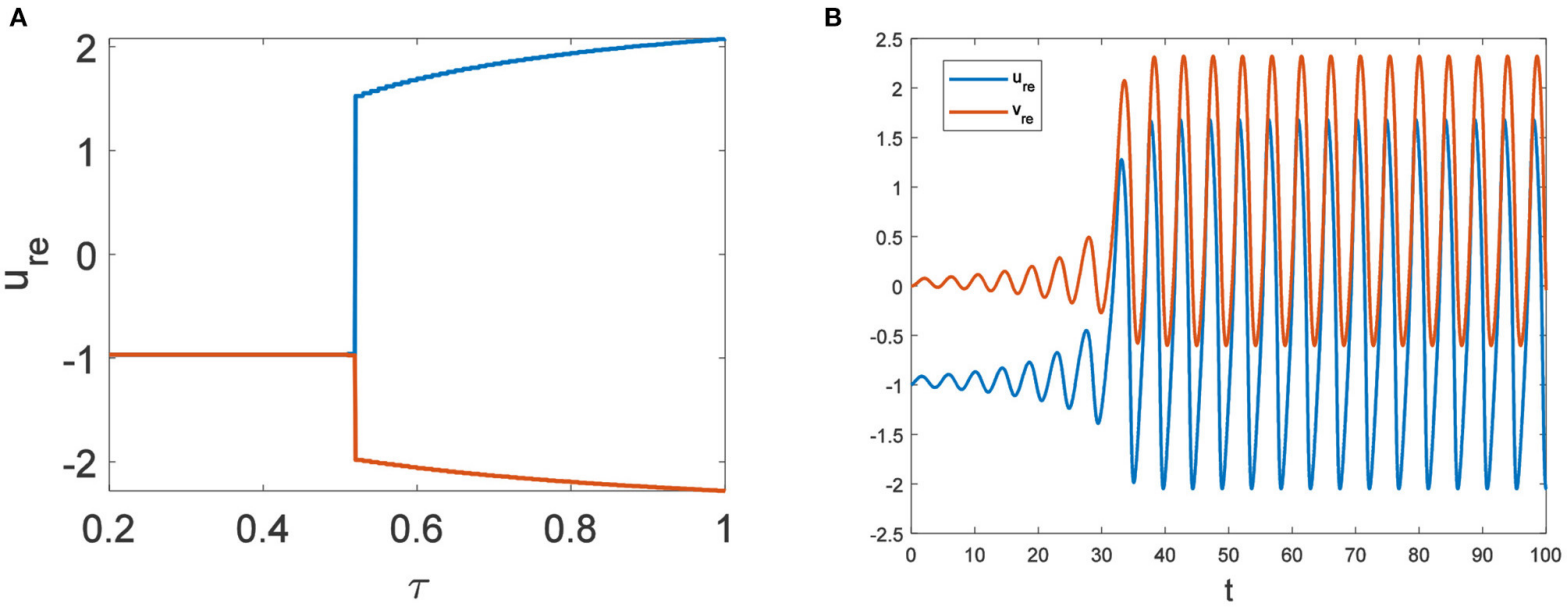
*D*_*u*_ = *D*_*v*_ = 0. **(A)** Bifurcation diagram about τ. The bifurcation point is τ = 0.5227. **(B)** The time series diagram with τ = 0.6.

**Figure 8:**
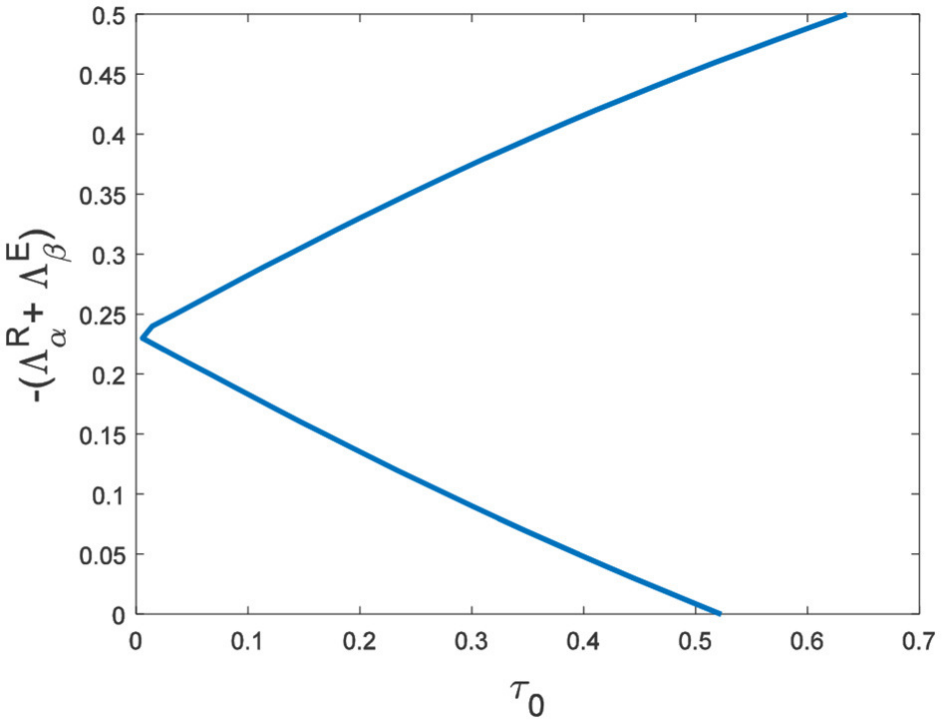
The relationship between τ_0_ and eigenvalues of *R*□*E*.

**Figure 9:**
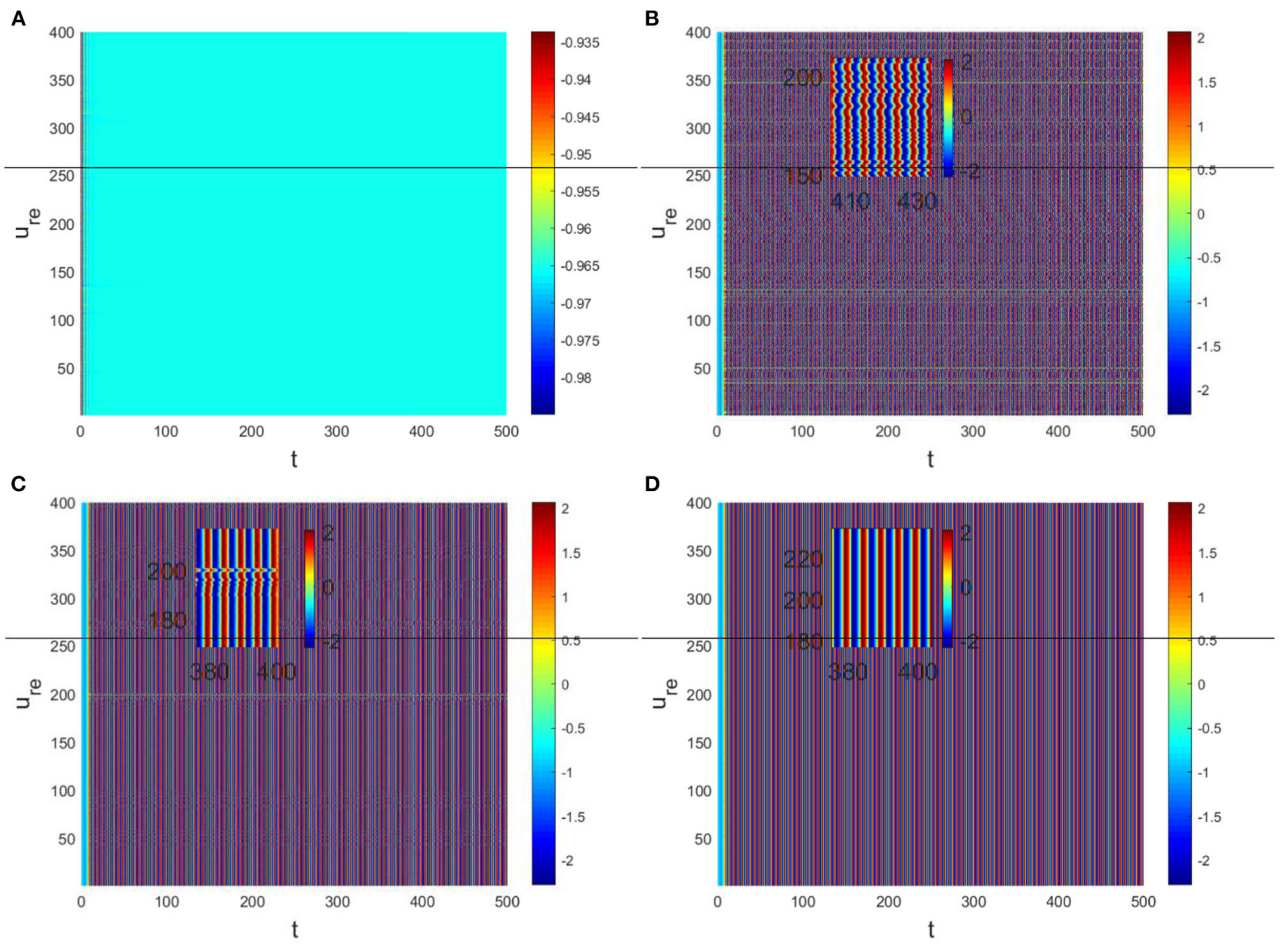
Pattern with *D*_*u*_ = 0.01, *D*_*v*_ = 8. **(A)** τ = 0.3, *p* = 0.01. **(B)** τ = 1, *p* = 0.01. **(C)** τ = 1, *p* = 0.1. **(D)** τ = 1, *p* = 0.3.

In the neural system, synapses can regulate the release of excitatory neurotransmitters of membrane potential or mediators through delayed feedback, so the response and transmission of signals will be delayed. Time delay affects the generation of bifurcation and phase synchronization between neurons, which affects the brain's memory function (27). Next, we explore the effect of time delay on neuronal activity. The transition of neurons from resting state to firing state is always accompanied by bifurcation behavior. Action potential exceeds the threshold when the time delay is greater than τ_0_ = 0.5227, regardless of the influence of the network (Figure 7). *c*_1_(0) = 0.021 − 0.2729*ι*, μ_2_ = −0.021, β_2_ = 0.042, *T*_2_ = 0.162 can be found in Equation (27). Namely, the system generates subcritical Hopf bifurcation (similarly, we can get the supercritical Hopf bifurcation). From Figure 8, the network will affect the value of τ_0_. In the study of the delayed neural network model, Zhao et al. (44) also found that the regulation of delay time can effectively control the formation of the pattern. Under the fixed network topology, the transmembrane current changes the membrane potential of neurons to different degrees with the increase of time delay. To more intuitively observe the collective behavior of neurons, we sorted 400 neuron nodes. When the delay time reaches 1, multiple neurons fire synchronously and participate in memory simultaneously (Figures 9A, B). It is found that the larger *p* is, the more obvious the synchronization phenomenon is (Figures 9B–D). Namely, short-term memory is relatively clear.

## 4. Conclusion

The brain is the most important organ in the human body, and its structure is very complex, so we have to simplify it when modeling. In this paper, we use the FHN model, which is simple but can describe the neuronal activity to explain the principle of short-term memory generation. The brain is a functional network that requires multiple neurons to work together for short-term memory. The brain regions responsible for specific tasks change their activity when the brain is storing memory (45). And pattern formation and selection can effectively detect collective behavior in excitable neural networks (27). Firstly, we establish the FHN model on the Cartesian product network and analyze the conditions of Turing instability. In the simulation, we found that short-term memory does not form when the probability of external stimulation and network connection is small. We test the robustness of the current results with Gaussian white noise and find that the system is robust when *np* < *O*(10^−5^). Short-term memory is formed when external stimuli, network connection probability, and noise reach a certain range. Because the pattern is not regular at this time, the short-term memory is blurred. Then we study the effect of time delay on short-term memory formation and find that short-term memory is formed when the delay time exceeds τ_0_. Of course, neuronal activity is not only related to external stimuli but the topology of the network itself. When *p* and delay time reach a certain degree, the cluster dynamic behavior appear, and the pattern shows periodic phenomenon. At this time, the brain forms a relatively clear short-term memory. These results provide a new way to explain the principle of memory formation.

## Data availability statement

The original contributions presented in the study are included in the article/supplementary material, further inquiries can be directed to the corresponding author.

## Author contributions

JS made contributions to the conception or design of mathematical model, supervision, and funding acquistition. JW made contributions to writing, editing–original draft, and formal analysis. All authors contributed to the article and approved the submitted version.
